# Research on Spatial Differences and Driving Effects of Ecological Well-Being Performance in China

**DOI:** 10.3390/ijerph19159310

**Published:** 2022-07-29

**Authors:** Shengyun Wang, Liancheng Duan, Shuwen Jiang

**Affiliations:** 1 Research Center of the Central China for Economic and Social Development, Nanchang University, Nanchang 330031, China; wangshengyun@163.com; 2 School of Economics and Management, Nanchang University, Nanchang 330031, China; duanliancheng0626@163.com; 3 School of Art, Nanchang University, Nanchang 330031, China

**Keywords:** ecological well-being performance, spatial differences, spatial polarization, LMDI method, driving effect

## Abstract

The essential requirement of sustainable development is to maximize economic prosperity and well-being while remaining within natural boundaries. This study focused on three aspects. First, a unique ecological well-being performance (EWP) evaluation model was developed by combining subjective and objective well-being indicators to assess China’s EWP from 2006 to 2018. Second, the evolution of spatial differences in China’s EWP was examined using the Dagum Gini coefficient and four spatial polarization indicators, from the perspective of eight economic regions. Third, we used the Logarithmic Mean Divisia Index (LMDI) method to decompose the driving factors of China’s EWP into four effects: economic, technical, objective well-being, and subjective well-being. Effective ways to promote the coordinated and sustainable enhancement of EWP in China were determined. The results showed that the overall level of EWP in China decreased from 2006 to 2018. The growth rate of China’s residents’ happiness index was not only slightly slower than the growth rate of the human development index but also significantly slower than the ecological footprint index per capita. The spatial differences of EWP in China were found to be expanding. Inter-regional differences were found to be the primary source of spatial differences in China’s EWP. Meanwhile, the capacity for sustainable development among provinces was further stretched, and, thus, the spatial polarization of China’s EWP tended to deepen. The importance of economic growth in boosting EWP cannot be overstated. China must actively encourage scientific and technological innovation, transition to a green development model, and raise human well-being in tandem with economic development. This study contributes to a scientific foundation and is a valuable reference for long-term and coordinated regional development in China and other emerging countries.

## 1. Introduction

China’s economy has grown dramatically in the last 40 years [1]. This economic boom has not only increased China’s wealth, job possibilities, and technical advancement but also considerably improved its people’s well-being [2]. According to the National Bureau of Statistics of China, the average life expectancy of the Chinese population increased from 68.2 to 77.93 years between 1978 and 2020 [3]. The gross enrollment rate in higher education increased from 0.26% to 54.4%, and the incidence of rural poverty decreased from 97.5% to 0.6%.

However, along with high economic growth, China also faces severe ecological degradation and over-consumption of resources [4], which reduces the environmental well-being of sustainable development and undermines overall human well-being [5]. According to a study by the Chinese Academy of Social Sciences [6], China is the world’s second-largest economy, but among 133 countries, it ranked ninth from the bottom in terms of eco-environmental competitiveness and second from the bottom in terms of air quality in 2012.

To achieve human-centered sustainable development, China must move its economy gradually toward high-quality development and its development model to “intensification, efficiency, greening, and well-being” [7]. The report of the 19th National Congress of the Communist Party of China emphasizes that the primary goal of development is to improve people’s well-being. Under the new development concept, improving the efficiency of natural resources and ecological inputs to the level of human well-being is an unavoidable choice to support China’s green transition and achieve sustainable development [8]. The concept of ecological well-being performance (EWP) first came from occidental countries and was proposed by Daly (1974) [9], who used this as an indicator to measure countries’ sustainable development. Zhu (2013) [10] introduced the concept of EWP to China and expressed EWP as the ratio of Human Development Index (HDI) to Ecological Footprint (EF). In essence, EWP measures eco-efficiency to enhance human well-being and is a broader extension of sustainable development [11,12]. Policymakers and academics focus on assessing EWP for coordinating economic, social, and environmental development and well-being improvement [13,14]. At the same time, compared with developed countries, such as the United States and European countries, China is a less developed country with a more severe situation of spatial differences and sustainable development. A detailed analysis of the evolution of spatial differences in China’s EWP and its driving effects can serve as a guide for promoting regional coordination and sustainable enhancement of EWP.

This study aimed to construct a comprehensive and accurate EWP evaluation model to quantitatively measure the EWP values at China’s regional and provincial levels. Then, we analyzed in-depth the sources of spatial differences and spatial polarization trends of EWP and clarified the policy priorities for China’s future coordinated regional development. Finally, the drivers of EWP were dissected to discover effective strategies for promoting long-term EWP improvement in China.

## 2. Literature Review

In recent years, considerable research around the world has focused on EWP and has achieved relatively fruitful results (Table 1). Early studies mainly focused on expanding the evaluation methods of EWP, which can be broadly divided into two types: the efficiency model and the ratio. The efficiency model method evaluates EWP by combining multiple input and output indicators and centers on calculating efficiency values based on the distance between each production unit point and frontier. For example, Dietz et al. (2009) [15] measured EWP for 135 countries based on the Stochastic Frontier Analysis (SFA) model. Bian et al. (2020) [16] used the super-efficiency slack-based measure (Super-SBM) model to evaluate the EWP of 30 provincial capitals in China.

The second method measures EWP by the ratio of well-being output to ecological consumption. Since Rees [24] introduced the EF in 1992, EF has been recognized as the definitive indicator of human ecological consumption. The characteristic and outstanding advantage of EF is the measurement of ecological consumption from the consumption side and in dimensions of both sources and sinks [25]. Well-being evaluation indicators can generally be classified as subjective, objective, and composite [8]. No other objective well-being indicator is as popular in academic and policy-making circles as the HDI [26], which was constructed on the theory of viable capacity of Sen (1989) [27], covers several countries and presents the advantages of being continuous in time and complete in content [28]. On this basis, Zhang et al. (2018) [8] applied the ratio of HDI to EF to measure EWP in 82 countries with populations above 10 million. In addition, common objective well-being indicators include the Physical Quality of Life Index (PQoL) [29] and Life Expectancy at Birth (LEB) [30]. Subjective well-being assessment indicators focus on individuals’ perceptions and feelings about the surrounding social environment [31], and commonly used factors include life satisfaction [32] and the happiness index [33]. In contrast, composite well-being indicators combine the advantages of both subjective and objective indicators, of which the Happy Life Years (HLY) [17] and the Happy Planet Index (HPI) [34] are typical. Common (2007) [17] applied the ratio of HLY to EF to measure the sustainable development in 143 countries.

Since then, relevant literature has gradually expanded from the evaluation method to research content, mainly focusing on analyzing spatial differences in EWP. Yao et al. (2020) [18] analyzed the spatial distribution characteristics of EWP in China through the spatial autocorrelation model and found a significant positive correlation. Wang et al. (2021) [19] used the σ and β convergence models to explore the spatial differences of EWP in eight economic regions in China. A convergence trend was found only for the southwest region, thus confirming that China’s EWP exhibits an imbalance with widening regional differences. Wang and Feng (2020) [20] used the Theil index to explore the spatial differences of EWP among the three major regions of east, central, and west China. The results indicated that inter-regional differences had a significant impact on the contribution of EWP.

In addition, the driving factors of EWP have been explored. Zhu and Zhang (2014) [21] decomposed EWP into the Economic Performance of Natural Consumption (EPNC) and Welfare Performance of Economic Output (WPEO) to explore its relationship with levels of well-being, natural consumption, and economic growth. Behjat and Tarazkar (2021) [22] used the Autoregressive Distributed Lag (ARDL) approach to explore the short- and long-term relationships between EWP and GDP per capita in Iran. Empirical results showed that GDP per capita had a significant and positive relation with EWP, but the effect of population and energy consumption was negative. Feng et al. (2019) [23] decomposed EWP into Industrial Structure Green Adjustment (ISGA) and Green Total Factor Productivity (GTFP) in a dynamic spatial panel model, which showed that both can effectively promote EWP in China. However, increasing GDP, expanding the scale of industrial agglomeration, and strengthening government intervention are not conducive to EWP.

Summarizing the existing literature, the EWP research has been expanded, but further improvements may be explored in the following areas. First, as the idea of “people-centered” development increasingly advances, measuring people’s happiness has become an integral part of assessing China’s EWP. However, objective well-being indicators are mainly used in constructing the evaluation model of EWP in China, and fewer studies select a combination of subjective and objective well-being indicators. Second, most existing research concentrates on assessing the spatial differences in China’s EWP by splitting into three primary regions: East, Central, and West. Not only is portraying the evolution of spatial differences in China’s EWP from many angles difficult, but the trend of spatial polarization is also rarely examined. Finally, existing studies have typically decomposed EWP into the ratio of GDP to EF and the ratio of HDI to GDP when exploring EWP drivers. Although this decomposition method links EWP with well-being, natural consumption, and economic growth, reflecting the direct impact of economic growth on China’s EWP is difficult. Moreover, subjective well-being indicators are not incorporated into the framework of EWP decomposition studies in China.

The following were the primary goals of this study. First, to accurately analyze China’s EWP, we first built a model based on the Comprehensive Well-being Index (CWI). Second, from the perspective of eight economic regions, the Dagum Gini coefficient and spatial polarization index were used to examine the evolution of spatial differences in China’s EWP, particularly the trend of spatial polarization. Third, using the Kaya Identity and LMDI method, we analyzed the driving effects of EWP in China with a unique paradigm and explored practical ways to promote sustainable development and coordinated regional development in China.

## 3. Materials and Methods

### 3.1. Methods

#### 3.1.1. EWP Evaluation Model

Referring to Common (2007) [17], we defined EWP as the ratio of human well-being level to ecological consumption. The level of human well-being was measured by HDI and residents’ happiness. Ecological consumption was measured by the EF per capita. According to Shi and Wang [35], the EF per capita is more informative than the total EF. Given that HDI is a unitless index with values in the range 0–1, while the values of EF per capita and residents’ happiness vary widely, we drew on the treatment of Zhang et al. (2018) [8] to normalize the latter values as follows:

(1) $$RHI=\frac{\ln\left(RH\right)-\ln\left(RH_{min}\right)}{\ln\left(RH_{max}\right)-\ln\left(RH_{min}\right)}$$

(2) $$EFI=\frac{\ln\left(EFpc\right)-\ln\left(EFpc_{min}\right)}{\ln\left(EFpc_{max}\right)-\ln\left(EFpc_{min}\right)}$$

where *RH* is the residents’ happiness; *RHI* is the residents’ happiness index; *EFpc* is the EF per capita; *EFI* is the EF index per capita. On this basis, we construct and express the EWP evaluation model as follows:

(3) $$EWP=\frac{CWI}{EFI}=\frac{HDI\times RHI}{EFI}$$

where *HDI* stands for human development index; *CWI* stands for composite well-being index, which is the product of *HDI* and *RHI*.

*HDI* is calculated using the method proposed by UNDP [28] (Table 2), which can be expressed as follows:

(4) $$HDI=\left(H_{1}+H_{2}+H_{3}\right)$$

where *H*_1_ represents the health index; *H*_2_ represents the education index, and *H*_3_ represents the income index. The closer the value of *HDI* is to 1, the higher the level of human development, and vice versa. The average life expectancy indicator was used to construct the health index. The education index was calculated as a weighted average of adult literacy and combined gross enrollment rates. The adult literacy rate was calculated as the percentage of the population aged 15 to 64 who can read and write in the total population aged 15 to 64, while the combined gross enrollment rate was calculated as the percentage of pupils aged 6 to 24 who were enrolled in school. The income index was calculated using gross domestic product (GDP) per capita adjusted by purchasing power parity (PPP) conversion factors.

The *RH* is the overall perception and evaluation of residents’ quality of life [36] and is generally measured by a questionnaire that assigns a base value to respondents’ self-reported level of happiness. The calculation method of *RH* in this study was to take the arithmetic mean of all samples of the residents’ happiness survey data.

The *EFpc* was calculated using the method proposed by Shi and Wang [35], expressed as follows:

(5) $$EFpc=\overset{n}{\underset{i}{\sum }}\left(r_{j}\times A_{i}\right)/N=\overset{n}{\underset{i}{\sum }}r_{j}\times \left(P_{i}/Y_{i}\right)/N$$

In Equation (5), *j* represents the *j*-th land-use type, including six types of arable land, forest land, grassland, building land, fishery land, and fossil fuel land; *r_j_* represents the equivalence factor of the *j*-th land use type (Table 3); *N* represents the total population; *A_i_* represents the area of productive biological land occupied by the *i*-th consumption item in discount (hm^2^); *P_i_* represents the total consumption of the *i*-th consumption item (kg). *Y_i_* represents the global average yield of the *i*-th consumption item (kg/hm^2^). The consumption items of arable land footprint include cereals, pulses, yams, oilseeds, cotton, tobacco, hemp, and sugar. However, since the consumption data of these eight items were difficult to obtain and their production and consumption were relatively close, the footprint was calculated using their yields [35]. The consumption of fisheries goods was used to compute the fishery land footprint. The footprint of forest land was calculated using the consumption of dried fresh fruits and vegetables, while that of grassland was calculated using the consumption of pork, beef and lamb, dairy, and poultry eggs. The building land footprint was calculated using electricity consumption. The fossil fuel land footprint was calculated using the consumption of eight energy sources: coal, coke, crude oil, gasoline, kerosene, diesel, fuel oil, and natural gas.

#### 3.1.2. Spatial Differences Measurement: Dagum Gini Coefficient Decomposition

The Gini coefficient is a useful metric for determining the extent of spatial differences. Based on this, Dagum Gini coefficient is an improved version that considers not only the main sources of spatial differences, but also samples cross-over and the distribution of subsamples [37]. The formula for calculating the Dagum Gini coefficient can be expressed as follows:

(6) $$G=\frac{\sum_{j=1}^{k}\sum_{h=1}^{k}\sum_{i=1}^{n_{j}}\sum_{r=1}^{n_{h}}\left|y_{ji}-y_{hr}\right|}{2\cdot \mu \cdot n^{2}}$$

where *μ* denotes the average value of EWP in all provinces; *n* denotes the number of provinces; *k* denotes the number of regions divided; *y_ji_*(*y_hr_*) denotes the level of EWP in province *i*(*r*) in region *j*(*h*); and *n_j_*(*n_h_*) denotes the number of provinces in region *j*(*h*).

According to the Dagum Gini coefficient decomposition method, *G* can be decomposed into the contributions of three components: intra-regional differences (*G_w_*), inter-regional differences (*G_b_*), and hypervariable density (*G_t_*), all of which satisfy *G= G_w_+ G_b_ + G_t_*. The *G_w_*, *G_b_*, and *G_t_* can be expressed as follows:

(7) $$G_{w}=\overset{k}{\underset{j=1}{\sum }}G_{jj}\cdot p_{j}\cdot s_{j}\;$$

(8) $$G_{b}=\overset{k}{\underset{j=2}{\sum }}\overset{j-1}{\underset{h=1}{\sum }}G_{jh}\cdot \left(p_{j}\cdot S_{h}+p_{h}\cdot S_{j}\right)\cdot D_{jh}$$

(9) $$G_{t}=\overset{k}{\underset{j=2}{\sum }}\overset{j-1}{\underset{h=1}{\sum }}G_{jh}\cdot \left(p_{j}\cdot S_{h}+p_{h}\cdot S_{j}\right)\cdot \left(1-D_{jh}\right)\;$$

(10) $$G_{jj}=\frac{\sum_{i=1}^{n_{j}}\sum_{r=1}^{n_{j}}\left|y_{ji}-y_{jr}\right|}{2\cdot \mu_{j}\cdot n_{j}^{2}}$$

(11) $$G_{jh}=\frac{\sum_{i=1}^{n_{j}}\sum_{r=1}^{n_{h}}\left|y_{ji}-y_{hr}\right|}{\left(\mu_{j}+\mu_{h}\right)\cdot n_{j}\cdot n_{h}}$$

(12) $$D_{jh}=\frac{d_{jh}-e_{jh}}{d_{jh}+e_{jh}}$$

(13) $$d_{jh}=\int_{0}^{\infty }dF_{j}\left(y\right)\int_{0}^{y}\left(y-x\right)dF_{h}\left(x\right)$$

(14) $$e_{jh}=\int_{0}^{\infty }dF_{h}\left(y\right)\int_{0}^{y}\left(y-x\right)dF_{j}\left(y\right)$$

(15) $$p_{j}=\frac{n_{j}}{n},\;s_{j}=\frac{n_{j}\cdot \mu_{j}}{n\cdot \mu }$$

where *G_jj_* denotes the spatial Gini coefficient of region *j*; *G_jh_* denotes the spatial Gini coefficient between spatial regions *j* and *h*; *D_jh_* denotes the interaction of EWP between regions *j* and *h*; *μ_j_* and *μ_h_* denote the mean values of EWP in regions *j* and *h*, respectively; *d_jh_* represents the mathematical expectation of all *y_ji_* – *y_hr_* > 0 in regions *j* and *h*; *p_jh_* represents the mathematical expectation of all *y_ji_* – *y_hr_* < 0 in regions *j* and *h*; and F(·) is the cumulative density function of regional EWP.

#### 3.1.3. Spatial Polarization Measurement: Wolfson Index, ER Index, EGR Index, and LU Index

At present, two types of indicators are mainly used to measure the degree of spatial polarization [38]. The first is the *W*-type index, among which the most commonly used is the Wolfson index. The second, the *ER*-type index, mainly includes *ER* index, *EGR* index, and *LU* index. To ensure the robustness of the conclusion, we selected the above four indices to analyze the trend of spatial polarization in China’s EWP.

The formula for calculating the Wolfson index can be expressed as follows:

(16) $$W=\frac{2\cdot \left(2T-G\right)}{m/\mu }$$

where *T* denotes the difference between the share of the number of low-level provinces and their share of EWP; *G* denotes the Gini coefficient; *m* denotes the median EWP of all provinces. Provinces with EWP lower than the national average were classified as the low-level group and vice versa as the high-level group. The value of the Wolfson index was between 0 and 1, and higher values indicated a higher degree of spatial polarization. In addition, the calculation of *ER* index, *EGR* index, and *LU* indices are expressed as follows:

(17) $$ER=Q\overset{k}{\underset{j=1}{\sum }}\overset{k}{\underset{h=1}{\sum }}p_{j}^{1+\alpha }\cdot p_{h}\cdot \left|\mu_{j}-\mu_{h}\right|$$

(18) $$ER=Q\overset{k}{\underset{j=1}{\sum }}\overset{k}{\underset{h=1}{\sum }}p_{j}^{1+\alpha }\cdot p_{h}\cdot \left|\mu_{j}-\mu_{h}\right|-\beta \left(G-G_{b}\right)\;$$

(19) $$LU=Q\overset{k}{\underset{j=1}{\sum }}\overset{k}{\underset{h=1}{\sum }}p_{j}^{1+\alpha }\cdot p_{h}\cdot {\left(1-G_{k}\right)}^{\beta }\cdot \left|\mu_{j}-\mu_{h}\right|\;$$

where *p_j_* and *p_h_* denote the share of the number of provinces in regions *j* and *h*, respectively; *α* is an arbitrary value between 0 and 1.6, usually 1.5 is chosen; *G_j_* denotes the Gini coefficient of region j; *Q* and *β* are adjustable normalization constants used to ensure that the spatial polarization index is between 0 and 1. Higher values of *ER*, *EGR*, and *LU* indices indicated higher spatial polarization of EWP.

#### 3.1.4. Decomposition Model of the Drivers of EWP Based on LMDI

In this study, the Kaya Identity was introduced to decompose the driving factors of EWP in China. According to the Kaya Identity [39], EWP can be extended from Equation (1), as follows:

(20) $$EWP=\frac{HDI\times RHI}{EFI}=GDP\times \frac{GDP}{EFI}\times \frac{RHI}{GDP}\times \frac{HDI}{GDP}=E\times T\times S\times O$$

where *GDP* stands for gross domestic product; *E* = *GDP* is an economic factor to reflect the level of China’s economic development; *T* = GDP/*EFI* is a technological factor to reflect the decoupling of China’s economic growth from ecological consumption; *S* = *RHI*/GDP and *O* = *HDI*/GDP are well-being factors, which are used to reflect the improvement of subjective and objective human well-being relative to economic development, respectively.

In this study, LMDI was used to quantify the driving role of each factor on EWP in China. Compared with other methods, LMDI has the advantage of not generating residuals and complete decomposition [40]. According to this model, the value of the change in EWP from the base period to year *t* can be expressed as △*EWP*, which carries out four effects: economic (*E_eff_*), technology (*T_eff_*), subjective well-being (*S_eff_*), and objective well-being (*O_eff_*).

(21) $$\Delta EWP=EWP_{t}-EWP_{0}=E_{eff}\times T_{eff}\times S_{eff}\times O_{eff}$$

(22) $$E_{eff}=\sum^{\;}\frac{\left(EWP_{t}-EWP_{0}\right)}{\left(\ln EWP_{t}-\ln EWP_{0}\right)}\times \ln\left(\frac{E_{t}}{E_{0}}\right)$$

(23) $$T_{eff}=\sum^{\;}\frac{\left(EWP_{t}-EWP_{0}\right)}{\left(\ln EWP_{t}-\ln EWP_{0}\right)}\times \ln\left(\frac{T_{t}}{T_{0}}\right)$$

(24) $$S_{eff}=\sum^{\;}\frac{\left(EWP_{t}-EWP_{0}\right)}{\left(\ln EWP_{t}-\ln EWP_{0}\right)}\times \ln\left(\frac{S_{t}}{S_{0}}\right)$$

(25) $$O_{eff}=\sum^{\;}\frac{\left(EWP_{t}-EWP_{0}\right)}{\left(\ln EWP_{t}-\ln EWP_{0}\right)}\times \ln\left(\frac{O_{t}}{O_{0}}\right)$$

If *E_eff_*, *T_eff_*, *S_eff_*, and *O_eff_* were positive, then the economic, technology, subjective well-being, and objective well-being effects could improve China’s EWP, which showed a positive driving effect; otherwise, a negative driving effect was observed.

### 3.2. Materials

#### 3.2.1. Division of Regions

Considering the availability of data, this study evaluated the EWP of China’s 30 provinces, except for Tibet, from 2006 to 2018 and analyzed the evolution of spatial differences and driving effects in China’s EWP from the perspective of eight economic regions. Referring to the studies of Ma et al. (2019) [41] and Wang et al. (2021) [19], the results of the eight economic regions were divided as follows (Table 4):

#### 3.2.2. Data Sources and Notes

Data sources were as follows: average life expectancy for the years before 2010 were from the Fifth Census of China [42], and for 2010 and beyond were from the Sixth Census of China [43]; PPP conversion factors were from the World Bank [44]; residents’ happiness data for 2006, 2008, 2010, and 2012 were from the Chinese General Social Survey (CGSS) survey project [45], and 2014, 2016, and 2018 residents’ happiness data for the years were from the China Family Panel Studies (CFPS) survey project [46]; Equivalence factors for six land categories were from Shi and Wang (2016) [35]; global average production data for various consumption items were from Wackernagel et al. (1999) [47]. All other data were from the National Bureau of Statistics of China from 2007 to 2019 [3].

We emphasize that the overall number of pupils aged 6 and up included those in special education, primary school, junior high school, secondary vocational school, ordinary high school, junior college, and undergraduate programs. In addition, in the CFPS survey project, respondents needed to answer the question “How happy do you feel?” in the range 0 to 10. In contrast, in the CGSS survey project, respondents needed to score by means of five levels: very happy, happy, general, unhappy, very unhappy. To make the data of the two survey projects more comparable, we carried out a 10-point assignment conversion for the data of the CGSS survey projects.

Matlab R2015b (MathWorks, Natick, Massachusetts, United States) was used to calculate the Dagum Gini coefficient and the spatial polarization index of EWP in China.

## 4. Results

### 4.1. Analysis of the Spatial and Temporal Evolution of EWP

#### 4.1.1. Time-Series Evolution of EWP in China

**Figure 1:**
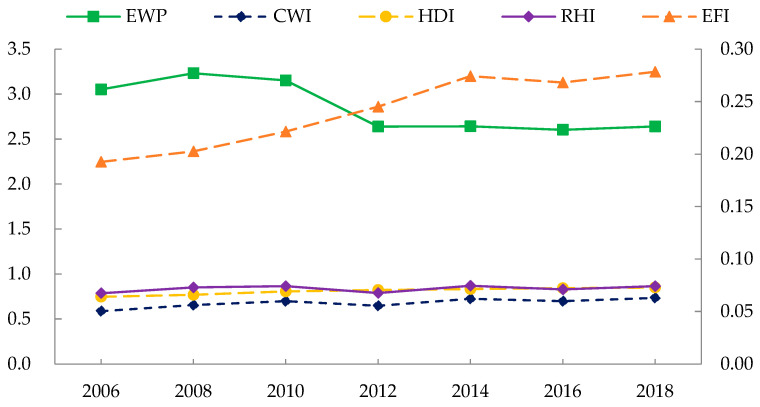
Temporal evolutionary trends of EWP and its components in China.

Based on Equation (1), EWP was expressed as *CWI* divided by *EFI*. Figure 1 shows the time-series evolutionary trend of China’s EWP and its components, with the right vertical axis reflecting the scale of *EFI*. As seen from the results, China’s overall EWP decreased from 3.052 in 2006 to 2.641 in 2018. Specifically, China improved its level of well-being to a certain extent. *HDI* improved from 0.747 in 2006 to 0.850 in 2018, moving from medium human development level to high human development level [48]. The growth rate of *RHI* in China was slightly lower than that of *HDI*, which also improved from 0.787 in 2006 to 0.865 in 2018. However, according to the calculations in this study, the fossil energy footprint per capita was the most significant component of China’s EF per capita, and China’s demand for coal-based fossil energy was particularly strong, leading to a significant upward trend in the *EFI*. Even though the growth rate of *EFI* slowed down after 2014, its average annual growth rate reached 3.088%. This figure was much higher than *CWI*, which explained the decline in China’s overall EWP from 2006 to 2018.

**Figure 2:**
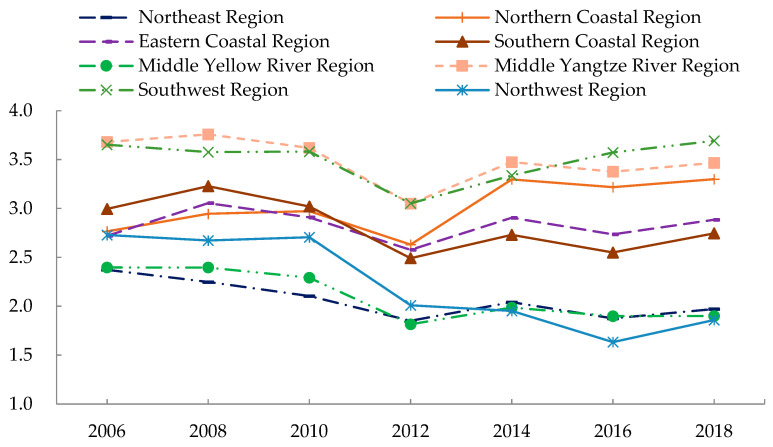
Temporal evolutionary trends of EWP in eight economic regions.

Figure 2 reflects the time-series evolution trend of EWP in the eight economic regions of China from 2006 to 2018. Among the eight economic regions, only the northern coastal region, the eastern coastal region, and the southwest region experienced an increase in EWP, which further reflected the trend of lower EWP in China as a whole. The middle Yangtze River region and the southwest region benefited from low ecological consumption and improved residents’ happiness. Their EWP far exceeded the national average, with annual average values of 3.495 and 3.489, respectively. However, the northwest region, the middle Yellow River region, and the northeast region had annual average values of only 2.223, 2.098, and 2.066, respectively, due to the damage to the ecological environment caused by crude development methods. The EWP of the northern, eastern, and southern coastal regions were close to the national average, with annual averages of 3.018, 2.826, and 2.823, respectively. The EWP of the northern coastal region increased dramatically after 2012 from 2.631 to 3.300 in 2018, with an average annual growth rate of 1.906%, thanks to Beijing’s progress in improving human well-being and ecological governance.

#### 4.1.2. Evolution of the Spatial Distribution of EWP in China

**Figure 3:**
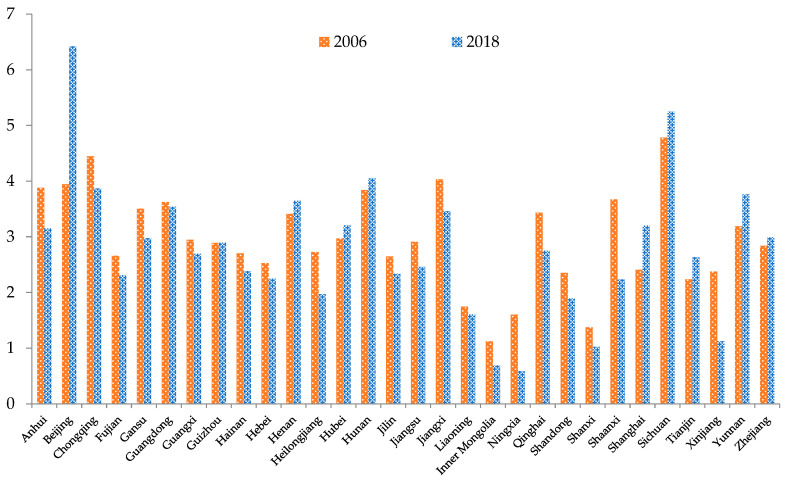
Comparison of the spatial distribution of EWP in China: 2006 and 2018.

As seen in Figure 3, the overall spatial structure of China’s EWP in 2006 was somewhat dispersed. Provinces with high EWP values were found in the southwest region, the northern coastal region, the southern coastal region, and the middle Yangtze River region, mainly including Sichuan, Chongqing, Beijing, Jiangxi, and Guangdong provinces. Provinces with lower EWP values were widely distributed in the northwest region, the middle Yellow River region, and the northeast region, mainly in Xinjiang, Inner Mongolia, Liaoning, Shandong, and Tianjin. In 2018, China’s EWP showed apparent block clustering. The EWP of provinces such as Guizhou and Yunnan gradually increased, while those of Jiangxi, Anhui, Guangdong, Shaanxi, Heilongjiang, and Jilin gradually decreased. The EWP in China showed the spatial pattern of “high values clustering in the southwest region, and low values spreading outward from the northeast region and the middle Yellow River region”.

### 4.2. Spatially Differences Analysis of EWP in China

Previous literature has revealed that China’s EWP has noticeable spatial differences. As a result, the present study used the Dagum Gini coefficient to quantify the spatial differences in China’s EWP and the spatial polarization index to investigate its spatial polarization trend.

#### 4.2.1. Spatial Differences of EWP in China

Table 5 shows the spatial Gini coefficient of China’s EWP year by year, increasing from 0.164 in 2006 to 0.237 in 2018. This finding indicated that the spatial differences in China’s EWP have been expanding. Specifically, the expansion can be divided into three stages: ① Slowly expanding period (2006–2010). Coastal areas had advantages in terms of economic development and technological level. They took the lead in implementing measures to eliminate backward production capacity and to promote equalization of basic public services to promote sustainable development, and EWP gradually increased. Thus, the spatial Gini coefficient of China’s EWP rose from 0.164 to 0.166, with an average annual growth rate of 0.101%; ② Rapid expanding period (2010–2016). Over this period, regions were increasingly focusing on long-term development and implementing a slew of policies aimed at “reducing pollution and improving well-being”. The sustainable development ability of each region increasingly widened, due to the influence of elements such as environmental governance level, economic growth stage, and other considerations. The spatial Gini coefficient of China’s EWP increased from 0.166 to 0.238, with an average annual growth rate of 3.048%; ③ Slowly shrinking period (2016–2018). The Chinese government proposed a new development paradigm in the 13th Five-Year Plan, which viewed regional collaboration as critical to the country’s overall “chess” development. The spatial difference of China’s EWP gradually decreased in this environment, and the spatial Gini coefficient decreased from 0.238 to 0.236, at an annual growth rate of 0.070%.

Similarly, this study measured the spatial Gini coefficient of EWP components in China by Equation (6). As seen in Table 5, the spatial Gini coefficients of HDI and RHI were relatively low, with annual average values of 0.028 and 0.031, respectively, indicating that China achieved a simultaneous spatial improvement in well-being. However, the spatial Gini coefficient of EFI rose to an annual average value of 0.243 and was growing. In this regard, the possible reason was the significant differences across Chinese regions in energy conservation and emission reduction norms, pollution control capacity, and critical function positioning, which were all still growing.

**Figure 4:**
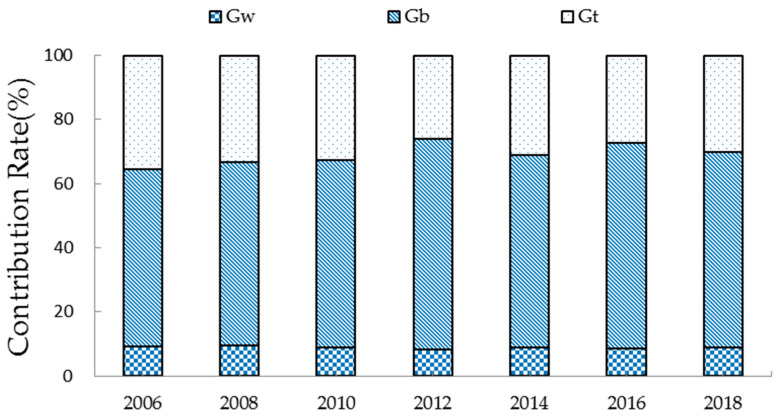
Sources of spatial differences in China’s EWP.

To reveal the sources of spatial differences of EWP, we measured the contribution rates of intra-regional differences (*G_w_*), inter-regional differences (*G_b_*), and hypervariable density (*G_t_*) based on Equations (7)–(9). Figure 4 shows that the contribution rate of *G_b_* had higher fluctuation, peaking at 65.70% in 2012, whereas the contribution rate of *G_w_* remained consistent at approximately 9.00%. The contribution of inter-regional differences was much more significant than intra-regional differences, implying that inter-regional differences affected the direction of spatial differences in China’s EWP, which was consistent with Wang et al. (2021) [13]. The takeaway was that reducing inter-regional differences is the key to mitigating spatial differences in China’s EWP.

Table 6 reflects the spatial Gini coefficient of EWP between the eight economic regions from 2006 to 2018. With annual average values of 0.296 and 0.281, respectively, the spatial Gini coefficient of EWP between the northeast region and the northwest region and between the northeast region and the southwest region were the greatest. This suggests that special attention must be paid to these regions, which have relatively significant inter-regional differences, to reduce spatial differences in China’s EWP. Furthermore, the spatial differences of EWP between the northern coastal region and the northwest region and between the northern coastal region and the southwest region were also significant, and the annual average values of spatial Gini coefficient were 0.272 and 0.267, respectively.

**Figure 5:**
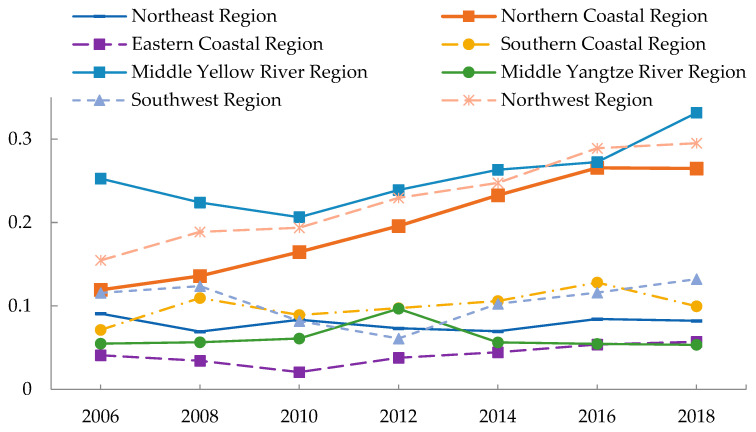
Trends of intra-regional differences of EWP in the eight economic regions.

Figure 5 depicts the evolution of spatial differences in EWP among the provinces within the economic region. From 2006 to 2018, the inter-provincial differences of EWP in the eastern coastal region were the smallest, while those in the middle Yellow River region were the largest. Regarding change trends, Beijing’s EWP increased dramatically from 2006 to 2018, widening the gap with Tianjin, Hebei, and Shandong. The result was a 6.865% average annual growth rate of spatial Gini coefficient in the northern coastal region. The spatial Gini coefficient in the northwest region increased by 5.531% per year during the same period, owing to considerable decreases in EWP in Ningxia and Xinjiang. The eastern coastal region, the southern coastal region, the middle Yellow River region, and the southwest region follow closely behind. Meanwhile, the spatial Gini coefficients of the northeast region and the middle Yangtze River region decreased by 0.202% and 0.827% per year, respectively. Notably, although the inter-provincial differences in EWP in the northeast region decreased, the price paid was that the EWP of three provinces, Liaoning, Jilin, and Heilongjiang, showed different degrees of decrease. As a result, while continuing to narrow the inter-regional differences of EWP, special attention must be directed to the severe problem of unsustainable economic and social development in the northeast region.

#### 4.2.2. Spatial Polarization of EWP in China

To further analyze whether the spatial polarization of EWP in China is deepening, we measured four spatial polarization indices of China’s EWP from 2006 to 2018. Table 7 reflects the evolution trend of the Wolfson index of EWP in China. Overall, the spatial polarization of EWP in China was increasing. The Wolfson index increased from 0.137 in 2006 to 0.179 in 2018, with an average annual increase of 2.253%. Specifically, from 2006–2016, the Wolfson index increased from 0.137 to 0.201 with an average annual growth rate of 3.246%, indicating that the spatial polarization of China’s EWP deepened during this time period. While from 2016–2018, the Wolfson index decreased from 0.201 to 0.179 with an average annual rate of 0.961%, indicating that the spatial polarization of China’s EWP decreased during this time period.

To better understand the spatial polarization of China’s EWP, we used the EWP of each province as a starting point and separated the 30 provinces into three groups based on 50% and 150% of the total average EWP in the year. Then, the provinces below 50% and above 150% of the average were considered to be bipolar provinces, while those between the two were considered to be intermediate provinces. Finally, three pivotal years were chosen for analysis: 2006, 2016, and 2018. Table 8 shows that the number of provinces in the two tiers increased year after year, and the proportion of all provinces increased from 10% in 2006 to 23.33% in 2018, confirming the preceding conclusion that China’s EWP became more spatially polarized. The implication was that there is a need to pay attention to the development of provinces with low sustainability, such as Inner Mongolia, Shanxi, Ningxia, and Xinjiang, and to ascertain how we can help improve their EWP by introducing targeted helping policies, thus, slowing the trend of China’s EWP spatial polarization.

**Figure 6:**
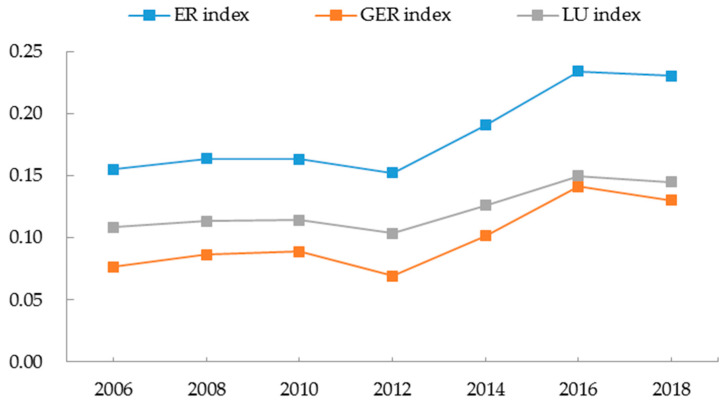
Trends in the evolution of the spatial polarization index of EWP in China.

In addition, Figure 6 shows that from 2006 to 2018, the *ER*, *EGR*, and *LU* indices all showed an overall increasing trend, indicating that the spatial polarization of EWP in China deepened. Furthermore, the evolution trends of the three polarization indices were nearly identical, increasing the robustness of the research conclusion. Numerically, the *ER* index was the largest of the three spatial polarization indices, followed by the *LU* index and the *GER* index.

### 4.3. Analysis of the Driving Effects of EWP in China Base on LMDI

#### 4.3.1. The Driving Effects of EWP in China

**Figure 7:**
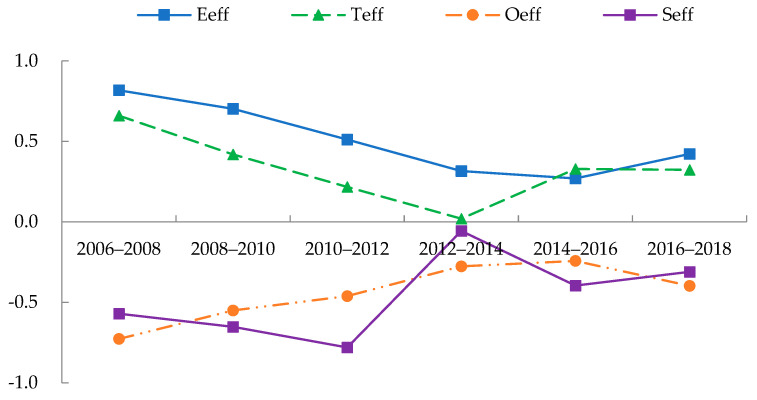
Driving effects of EWP in China: 2006−2018.

Following Equations (21)–(25), the driving effects of China’s EWP could be calculated as shown in Figure 7. Economic and technological effects were the positive drivers of EWP in China from 2006 to 2018, while the objective and subjective well-being effects were negative drivers. Together, the four drivers contributed to the lower overall EWP in China. Specifically, the economic effect had the highest average annual value, indicating that economic growth played the most significant role in boosting China’s EWP. However, this effect was weakening with the gradual slowdown of China’s economic growth. The technical effect was also a positive driving force that produced a vital boost to China’s EWP. Technological advancements not only helped improve resource usage efficiency and boosted China’s green economic development but also improved human well-being in areas such as the environment, education, and healthcare. Notably, the technology effect also gradually waned during the study period, which indicated the lack of a sustained enhancement momentum in China’s EWP.

The objective well-being effect was a negative driving effect, constantly pulling down China’s EWP, which indicated that the objective well-being generated by China’s economic growth was low. China still had more room for progress in public education, infrastructure, health care and other livelihood aspects and still faced the challenge that the level of human development could not increase in tandem with economic growth. However, the objective well-being effect also showed a trend of continuous improvement. With the completion of a moderately prosperous society, China’s traditional development model of “emphasizing economic development at the expense of growth in well-being growth” was transformed.

The subjective well-being effect was also a negative driving force with the lowest annual average effect value, which indicated that China’s EWP was mainly constrained by the inability of residents’ happiness to increase in tandem with the level of economic development. In particular, from 2006 to 2010, China’s GDP grew at an average annual rate of 11.2%. Still, social development mainly pursued improvement in material living standards and paid far less attention to subjective well-being. This approach led to ecological damage and inhibited the enhancement of residents’ happiness, and ultimately the subjective well-being effect continued to diminish. In recent years, China has promoted people’s well-being and all-round development as the starting and ending points of work, investing more of its economic output in the construction of livelihoods and emphasizing whether people are happy and satisfied with their living environment. Thus, the growth rate of RHI is gradually catching up with the growth rate of EFI, and the effect of subjective well-being has improved significantly.

The consequence is that to achieve sustainable development, China must continue to foster scientific and technical innovation, the transition toward a green development model with innovation as the engine, and decouple economic growth from ecological consumption [10]. At the same time, China must strengthen its investment in people’s lives and continue to improve residents’ happiness to offer a strong impetus for EWP improvement.

#### 4.3.2. Driving Effects of EWP in Eight Economic Regions

The study further analyzed the driving effects of EWP in the eight economic regions from 2006–2010, 2010–2014 and 2014–2018. Table 9 shows the decomposition results using the LMDI model.

(1) Economic effect. The economic effect of EWP in all eight economic regions from 2006 to 2018 showed a positive driving effect but continued to weaken. Specifically, the economic effect of the southwest region was the strongest, with a value of 4.765, which played a more significant role in promoting EWP. The economic effects of the northeast and middle Yellow River regions were the weakest, especially in the northeast region, where the economic effect was 0.908 from 2006 to 2010 and decreased to 0.107 from 2014 to 2018. The EWP in the region also decreased significantly.

(2) Technical effect. The technical effect of EWP in all eight economic regions revealed a positive driving effect from 2006 to 2018. The southwest region’s technical effect was strengthening, while other regions’ technical effect was weakening. Specifically, the technical effect of the middle Yangtze River region was the strongest before 2010, and the technical effect of the southwest region increased to the top of the eight economic regions after 2010. The technical effects of the northeast region, the middle Yellow River region, and the northwest region were the weakest. These three regions needed to accelerate the promotion of scientific and technological innovation and the improvement of resource utilization efficiency to reverse the downward trend of EWP in the region.

(3) Objective well-being effect. The objective well-being effect of all eight economic regions exhibited a dampening effect on EWP from 2006 to 2018, indicating that China was inefficient in translating economic growth into levels of human development. However, the objective well-being effect of the eight economic regions increased to varying degrees, which indicated that the achievements of China’s economic development had begun to be reflected in HDI. Notably, the objective well-being effects of the coastal region, the middle Yangtze River region, and the southwest region improved slowly, indicating that the improvement of human development still lagged behind economic growth and had not significantly changed.

(4) Subjective well-being effect. The subjective well-being effect of all eight economic regions dampened EWP from 2006 to 2018, indicating that the improvement of residents’ happiness in these regions also lagged behind economic growth. Not only was the objective well-being effect in the northern coastal region slower to improve, but the subjective well-being effect was also weakening. The northern coastal region must pay special attention to improving the level of human well-being, enhancing residents’ happiness and actively promoting the equalization of basic public services in the region.

## 5. Discussion

EWP is uniquely positioned to measure the level of sustainable development and to coordinate economic, environmental, and social development. To comprehensively and accurately measure the level of sustainable development, this study incorporated subjective well-being indicators into the evaluation model of China’s EWP, which proved necessary. Bian et al. (2019) [49] and Feng et al. (2019) [23], considering only objective levels of well-being, arrived at the conclusion that the overall EWP in China was gradually improving. In this study, after considering residents’ happiness, the empirical results showed that the overall EWP in China declined from 2006 to 2018, consistent with Yao et al. (2020) [18]. On the one hand, this was because the growth rate of China’s *EFI* was faster than indicators such as resource consumption or environmental pollution in other studies. On the other hand, the growth rate of residents’ happiness was still very low. China’s economy is gradually shifting from rapid growth to high-quality development, and the government must focus on limiting the negative consequences of economic expansion on ecology and the environment to improve residents’ happiness [19]. In future research, it is necessary to expand the application of subjective well-being indicators further to provide an up-to-date research perspective and analytical tools for sustainable development in China.

This study analyzed the spatial differences of EWP in China by dividing it into eight economic regions. Compared with the division method of the three major regions of East, Central and West, such a division method can portray the spatial differences of EWP in China in a more detailed way. The results indicated the relatively apparent spatial differences in EWP between the southwest and northeast regions and the northwest and northeast regions. China must break administrative barriers across regions and create a cross-regional sustainable development partnership framework [13]. Moreover, the capacity for sustainable development is further stretched between provinces. This has led to a deepening spatial polarization of EWP in China. To avoid further deepening of spatial polarization induced by imbalanced resource allocation, China must boost top-level design and provide policy to provinces with weaker sustainable development capacity.

In addition, for China, economic growth is the primary goal of development. This study examined the direct effect of economic growth on EWP in China, and such an approach is rich in significance. Although economic progress has resulted in pollution and ecological degradation, its contribution to human well-being cannot be overlooked. Meanwhile, this study refered to the decomposition of EWP by Zhu and Zhang (2014) [21], which placed higher demands on developing countries, including China, to achieve sustainable development. For developing countries that lag behind the United States and developed countries in Europe, in terms of economic development level and technological level, it is necessary to actively encourage technological innovation and green economic transformation to decouple economic growth from environmental consumption. Developing countries cannot only pursue economic growth, but must promote the relative development of the human development level and residents’ happiness to promote EWP enhancement. In addition, this study explored the driving effects of EWP in eight economic regions to provide a methodological reference for policymakers in other developing countries to formulate targeted and sustainable regional development policies.

## 6. Conclusions

First, overall EWP in China showed a decreasing trend from 2006 to 2018. Among the eight economic regions, the EWP of the northern coastal region, the eastern coastal region, and the southwest region showed an increasing trend. In contrast, the other regions showed different degrees of decrease. The EWP in the middle Yangtze River region and the southwest region is far above the national average, while that in the northeast region is the lowest. The EWP in China shows the spatial pattern of “high values clustering in the southwest region, and low values spreading outward from the northeast region and the middle Yellow River”.

Second, the spatial differences of EWP in China are widening, with inter-regional differences being the primary cause of these variances. The problem of spatial differences in EWP between the southwest and northeast regions and between the northwest and northeast regions is most prominent. The inter-provincial differences in EWP in the middle Yellow River region are the largest. Although inter-provincial differences in EWP decrease within the northeast region, economic and social development tends to be unsustainable.

Third, the spatial polarization of EWP in China deepened from 2006 to 2018. This is manifested by a significant decrease in the number of provinces with EWP levels in the middle strata. Provinces such as Inner Mongolia, Shanxi, Ningxia, and Xinjiang are further pulled apart by provinces with high EWP levels. China must pay special attention to the development of those provinces lagging in sustainable development.

Fourth, economic and technical effects have been driving China’s EWP, while objective and subjective well-being effects have been pulling it down. EWP progress in China is severely hampered by the country’s slow rate of improvement in well-being relative to economic development. The southwest region has made significant progress in terms of economic development and technical level, which has aided in EWP improvement. The technical effect is weakest in the northeast region, the Middle Yellow River region, and the northwest region. The objective well-being effect in the coastal region, the middle Yangtze River region, and the southwest region are slower to improve. The subjective well-being effect in the northern coastal region is still weakening.

## Figures and Tables

**Table 1:** Summary of studies related to EWP.

Category	Model	Author	Objective Area
EWP evaluation	SFA	Dietz et al. (2009) [15]	135 countries
	Super-SBM	Bian et al. (2020) [16]	30 Provincial Capital Cities in China
	The ratio of HDI to EF	Zhang et al. (2018) [8]	82 countries with populations above 10 million
	The ratio of HLY to EF	Common (2007) [17]	143 countries
Spatial differences in EWP	Spatial autocorrelation model	Yao et al. (2020) [18]	30 provinces in China
	σ and β convergence models	Wang et al. (2021) [19]	8 economic regions in China
	Theil index	Wang and Feng (2020) [20]	30 provinces in China
The driving factors of EWP	Equality decomposition	Zhu and Zhang (2014) [21]	124 countries
	Autoregressive distributed lag model	Behjat and Tarazkar (2021) [22]	Iran
	Dynamic spatial panel model	Feng et al. (2019) [23]	30 provinces in China

**Table 2:** HDI calculation.

Sub-Indices	Indicators	Calculation Method
Health Index (*H*_1_)	*H*: Average life expectancy	$H_{1}=\frac{H-25}{85-25}$
Education Index (*H*_2_)	*E*_1_: Adult literacy rate *E*_2_: Gross enrollment rate	$H_{2}=\frac{2}{3}E_{1}+\frac{1}{3}E_{2}$
Income Index (*H*_3_)	*W*: PPP conversion factors adjusted GDP per capita	$H_{3}=\frac{\ln W-\ln100}{\ln40000-\ln100}$

**Table 3:** Equivalence factors for the six land types.

Factor	Arable Land	Woodland	Grassland	Building Site	Fishery Land	Fossil Fuel Land
Equivalence factors	2.21	1.34	0.49	2.21	0.20	1.34

**Table 4:** Division of eight economic regions.

Region Name	Provinces Included
Northeast Region	Heilongjiang, Jilin, Liaoning
Northern Coastal Region	Beijing, Tianjin, Hebei, Shandong
Eastern Coastal Region	Shanghai, Jiangsu, Zhejiang
Southern Coastal Region	Fujian, Guangdong, Hainan
Middle Yellow River Region	Shanxi, Inner Mongolia, Henan, Shaanxi
Middle Yangtze River Region	Anhui, Jiangxi, Hubei, Hunan
Southwest Region	Guangxi, Chongqing, Sichuan, Guizhou, Yunnan
Northwest Region	Ningxia, Gansu, Qinghai, Xinjiang

**Table 5:** Dagum Gini coefficient of EWP and its components in China.

Indicator	2006	2010	2012	2014	2016	2018
EWP	0.1637	0.1663	0.1988	0.2050	0.2381	0.2366
HDI	0.0390	0.0249	0.0233	0.0226	0.0244	0.0234
RHI	0.0227	0.0314	0.0308	0.0362	0.0340	0.0270
EFI	0.2188	0.2167	0.2441	0.2554	0.2600	0.3001

**Table 6:** Spatial differences in EWP among the eight economic regions from 2006 to 2018.

Region	G_jh_	Region	G_jh_	Region	G_jh_	Region	G_jh_
1–2	0.104	2–3	0.185	3–5	0.173	4–8	0.205
1–3	0.187	2–4	0.127	3–6	0.221	5–6	0.176
1–4	0.145	2–5	0.147	3–7	0.260	5–7	0.210
1–5	0.166	2–6	0.223	3–8	0.262	5–8	0.213
1–6	0.242	2–7	0.267	4–5	0.122	6–7	0.226
1–7	0.281	2–8	0.272	4–6	0.180	6–8	0.214
1–8	0.296	3–4	0.171	4–7	0.221	7–8	0.243

Note: 1, 2, 3, 4, 5, 6, 7 and 8 represent the northeast region, the northern coastal region, the eastern coastal region, the southern coastal region, the middle Yellow River region, the middle Yangtze River region, the southwest region, and the northwest region, respectively.

**Table 7:** Spatial polarization trend of EWP in China: based on the Wolfson index.

Year	2006	2008	2010	2012	2014	2016	2018
Wolfson Index	0.137	0.137	0.136	0.153	0.172	0.201	0.179

**Table 8:** Spatial polarization of EWP in China in 2006, 2016 and 2018.

Category	2006	2016	2018
Bipolar provinces (≧Average value × 150%)	Sichuan	Beijing, Hunan, Sichuan, and Chongqing	Beijing, Hunan, Sichuan
Bipolar provinces (≤Average value × 50%)	Inner Mongolia, Shanxi	Inner Mongolia, Ningxia Xinjiang	Inner Mongolia, Shanxi, Ningxia, Xinjiang
Intermediate provinces	Other provinces	Other provinces	Other provinces

**Table 9:** Driving effects of EWP in eight economic regions: 2006–2018.

Region	Period	Eeff	Teff	Oeff	Seff	△EWP
Northeast Region	2006–2010	0.908	0.403	−0.747	−0.833	−0.268
	2010–2014	0.539	0.343	−0.521	−0.424	−0.062
	2014–2018	0.107	0.060	−0.124	−0.114	−0.071
	2006–2018	1.586	0.830	−1.427	−1.390	−0.401
Northern Coastal Region	2006–2010	1.060	0.816	−0.932	−0.738	0.207
	2010–2014	0.546	0.720	−0.488	−0.454	0.324
	2014–2018	0.683	0.776	−0.605	−0.850	0.003
	2006–2018	2.259	2.276	−1.999	−2.003	0.534
Eastern Coastal Region	2006–2010	1.151	0.898	−1.017	−0.843	0.189
	2010–2014	0.625	0.531	−0.566	−0.594	−0.004
	2014–2018	0.748	0.707	−0.662	−0.813	−0.020
	2006–2018	2.473	2.093	−2.201	−2.201	0.165
Southern Coastal Region	2006–2010	1.431	0.937	−1.249	−1.094	0.025
	2010–2014	0.817	0.511	−0.737	−0.880	−0.289
	2014–2018	0.725	0.690	−0.639	−0.760	0.015
	2006–2018	2.971	2.155	−2.625	−2.750	−0.249
Middle Yellow River Region	2006–2010	0.867	0.558	−0.706	−0.801	−0.105
	2010–2014	0.471	0.223	−0.439	−0.499	−0.305
	2014–2018	0.428	0.305	−0.389	−0.414	−0.088
	2006–2018	1.788	1.093	−1.566	−1.713	−0.497
Middle Yangtze River Region	2006–2010	2.147	1.426	−1.793	−1.842	−0.062
	2010–2014	1.266	0.916	−1.189	−1.137	−0.144
	2014–2018	0.970	0.859	−0.866	−0.970	−0.008
	2006–2018	4.458	3.264	−3.926	−4.009	−0.214
Southwest Region	2006–2010	1.973	1.184	−1.582	−1.644	−0.070
	2010–2014	1.419	1.158	−1.317	−1.505	−0.244
	2014–2018	1.135	1.303	−1.008	−1.076	0.355
	2006–2018	4.765	3.860	−4.124	−4.459	0.041
Northwest Region	2006–2010	1.374	0.379	−1.094	−0.683	−0.023
	2010–2014	0.797	0.025	−0.723	−0.879	−0.754
	2014–2018	0.374	0.286	−0.349	−0.382	−0.093
	2006–2018	2.421	0.729	−2.074	−1.945	−0.870

## Data Availability

The data presented in this study are available from the corresponding author.
